# Apparent Temperature and Air Pollution vs. Elderly Population Mortality in Metro Vancouver

**DOI:** 10.1371/journal.pone.0025101

**Published:** 2011-09-28

**Authors:** Goran Krstić

**Affiliations:** Fraser Health, Environmental Health Services, New Westminster, British Columbia, Canada; Centre de Recherche Public de la Santé (CRP-Santé), Luxembourg

## Abstract

**Background:**

Meteorological conditions and air pollution in urban environments have been associated with general population and elderly mortality, showing seasonal variation.

**Objectives:**

This study is designed to evaluate the relationship between apparent temperature (AT) and air pollution (PM_2.5_) vs. mortality in elderly population of Metro Vancouver.

**Methods:**

Statistical analyses are performed on moving sum daily mortality rates vs. moving average AT and PM_2.5_ in 1-, 2-, 3-, 5-, and 7-day models for all seasons, warm temperatures above 15°C, and cold temperatures below 10°C.

**Results:**

Approximately 37% of the variation in all-season mortality from circulatory and respiratory causes can be explained by the variation in 7-day moving average apparent temperature (r^2^ = 0.37, p<0.001). Although the analytical results from air pollution models show increasingly better prediction ability of longer time-intervals (r^2^ = 0.012, p<0.001 in a 7-day model), a very weak negative association between elderly mortality and air pollution is observed.

**Conclusions:**

Apparent temperature is associated with mortality from respiratory and circulatory causes in elderly population of Metro Vancouver. In a changing climate, one may anticipate to observe potential health impacts from the projected high- and particularly from the low-temperature extremes.

## Introduction

Ambient air temperature is a recognized seasonal factor associated with mortality in the general population, particularly in the vulnerable elderly [1]–[5]. Heat waves are associated with short-term (1–3 day) spikes in mortality, followed by lower than average mortality rates in the subsequent days. There is evidence in the published literature for the presence of a lag time between the exposure and an effect, showing that lags of 0 to 3 days during heat waves provide the best prediction of mortality rates [5], [6], [7]. On the other hand, under cold winter conditions, mortality rates gradually increase with less pronounced short-term effects. Longer lag times appear to be better in predicting mortality during cold spells [2], [8], [9].

A skewed V-shaped relationship has been observed for ambient air temperature vs. mortality [5]. Depending on the geographical area, temperature extremes outside a thermal comfort zone of approximately 15° to 26°C (60 to 80°F) may lead to an elevated stress and increased population mortality [4], [5], [10].

Regarding air pollution, epidemiological studies indicate an association between airborne particulate matter (PM) and mortality in urban environments [11]–[16]. Lag periods of 0 to 7 days between the exposure to air pollution and the time of death have been considered in the published literature, where longer lags appear to be better predictors for respiratory and shorter lags for cardiovascular mortality [17]–[20].

A variation in population characteristics and environmental/meteorological parameters may affect our ability to predict and quantify accurately short- or long-term health effects from air pollution and/or outdoor temperature extremes. This study is designed to evaluate the relationship between the seasonal variation in apparent temperature (AT) and air pollution (PM_2.5_) vs. circulatory and respiratory (C&R) mortality in Metro Vancouver elderly population.

## Materials and Methods

The geographical region of interest for this study includes Fraser Health (FH) and Vancouver Coastal Health (VCH) service delivery areas of Metro Vancouver, British Columbia (BC). The mortality in relation to particulate matter air pollution with aerodynamic diameter <2.5 µm (PM_2.5_) and apparent temperature is studied in the elderly population (i.e., >65 years of age) for the period from January 2004 to December 2006. Although this study has been approved by Fraser Health Research Ethics Board (FHREB) as part of an ethics committee review process (FHREB Reference No. 2008-022), no human subjects or animals were involved. Therefore, the paper does not require an ethics statement or a written consent from the patients.

### Mortality data

Daily mortality data are obtained from the British Columbia Vital Statistics Agency, Ministry of Health. The World Health Organization (WHO) International Classification of Diseases (ICD-10) scheme is used to extract the diseases of the respiratory (ICD-10 codes: J00–J06, J13, and J15–J99) and the circulatory system (ICD-10 codes: I00–I99). In an effort to control for a potentially strong confounding effect of seasonal influenza outbreaks on daily mortality [2], [4], [18], identified cases of influenza (ICD-10 codes: J09–J11), viral pneumonia (ICD-10 code J12), and Haemophilus influenza (ICD-10 code J14) are excluded from the study. Mortality rates per 100,000 are calculated using the total elderly population in the studied area for the years 2004, 2005, and 2006.

### Ambient air quality and meteorological data

Daily mean airborne PM_2.5_ concentrations (µg/m^3^), air temperature (°C), relative humidity (%) and wind speed (m/s) data are obtained from the network of 13 monitoring stations of Metro Vancouver. The values for apparent temperature (AT), as a measure of perceived outdoor air temperature, are calculated using the method as described by Steadman in the norms of apparent temperature [21]. The following formula is applied in the calculation: AT = Ta+0.33 _*_ e – 0.70 _*_ ws – 4.00, where ***Ta*** is air temperature (°C), ***e*** water vapour pressure or humidity (hPa), and ***ws*** wind speed (m/s) at an elevation of 10 meters. The vapour pressure ***e*** is calculated from air temperature and relative humidity using the equation: e = rh/100 _*_ 6.105 _*_ exp(17.27 _*_ Ta/(237.7+Ta)), where ***rh*** is relative humidity (%). Kunst et al. (1994) suggest that Steadman's AT is a better measure of human response to wind-chill related stress in cold season than simple ambient air temperature or other thermal indices [22].

### Statistical analysis

Descriptive statistics and scatter plot analyses revealed no evidence of non-normality in the distribution of mortality, AT and PM_2.5_ data allowing the use of linear regression. Statistical analyses are performed on moving sum daily mortality rates for circulatory and respiratory causes vs. moving average AT and PM_2.5_ in 1-, 2-, 3-, 5-, and 7-day models. The strength, direction, and statistical significance of Pearson's correlation coefficients (***r***) are observed and the AT and PM_2.5_ evaluated in terms of their ability to explain the variation in the response variable (i.e., mortality from circulatory and respiratory causes) by calculating the coefficients of determination (***r^2^***) for all seasons, warm temperatures above 15°C, and cold temperatures below 10°C.

## Results

**Figure 1:**
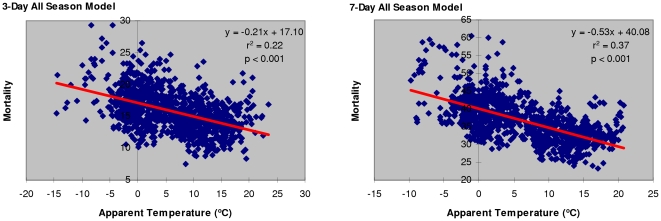
Mortality per 100K elderly population from circulatory and respiratory causes associated with apparent temperature (3-day and 7-day all-season models).

**Figure 2:**
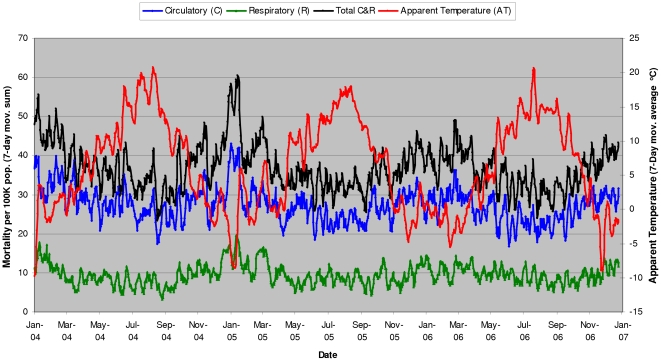
Elderly mortality in Metro Vancouver vs. apparent temperature - seasonal cycles (2004–2006).

Linear regression analyses show that all-season mortality in Metro Vancouver elderly population is associated with apparent temperature (**Table 1** **,** **Figure 1**). Longer time-interval models appear to be better in predicting temperature-related mortality, where up to 37% of the variation in all-season mortality from circulatory and respiratory causes (C&R) can be explained by the variation in 7-day moving average apparent temperature (r^2^ = 0.37, p<0.001). Seasonal cycles in elderly mortality vs. apparent temperature are observed, with up to 2-fold higher mortality rates per 100,000 elderly population in winter than in summer periods (**Figure 2**).

**Table 1:** Linear regression estimates of all-season mortality from circulatory and respiratory causes in Metro Vancouver elderly population associated with apparent temperature.

All-SeasonLinear Regression Coefficient/Variable‡	1-Day Model	2-Day Model	3-Day Model	5-Day Model	7-Day Model
Y-axis Intercept	5.69±0.06***	11.39±0.09***	17.10±0.11***	28.57±0.16***	40.08±0.19***
AT (°C)	−0.07±0.01***	−0.14±0.01***	−0.21±0.01***	−0.37±0.02***	−0.53±0.02***
Observations Count	1096	1095	1094	1092	1090
Coefficient of Determination (**r^2^**)	0.10	0.17	0.22	0.30	**0.37**

***p<0.001.

‡ Plus-minus values are linear regression coefficients and standard errors (i.e., ±SE).

AT – Apparent Temperature.

**Figure 3:**
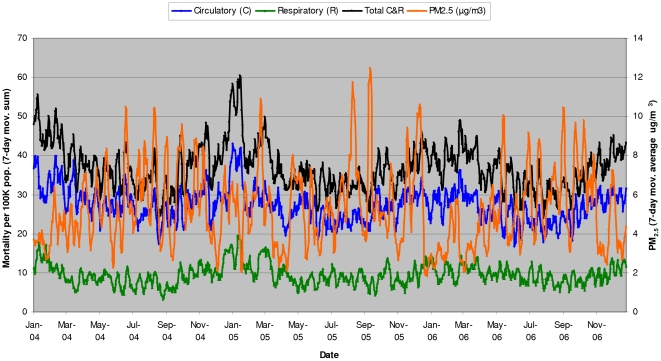
Elderly mortality in Metro Vancouver vs. air pollution (PM_2.5_) - seasonal cycles (2004–2006).

Air pollution (PM_2.5_) in Metro Vancouver is not a reliable predictor of mortality in 3-, 5-, and 7-day models (**Table 2**). Shorter interval models could not be used in the analysis due to the observed evidence of non-normality in the distribution of 1- and 2-day PM_2.5_ data. Although the analytical results from air pollution models show increasingly better prediction ability of longer time-intervals (r^2^ = 0.012, p<0.001 in a 7-day model), the correlation between elderly mortality and air pollution appears to be very weak and, paradoxically, negative (i.e., lower levels of air pollution in winter are associated with higher mortality rates (**Figure 3**)).

**Table 2:** Linear regression estimates of all-season mortality from circulatory and respiratory causes in Metro Vancouver elderly population vs. air pollution (PM_2.5_).

All-SeasonLinear Regression Coefficient/Variable‡	3-Day Model	5-Day Model	7-Day Model
Y-axis Intercept	16.25±0.22***	27.29±0.36***	38.48±0.50***
Air Pollution (PM_2.5_) (µg/m^3^)	−0.09±0.04*	−0.19±0.06**	−0.33±0.09***
Observations Count	1094	1092	1090
Coefficient of Determination (**r^2^**)	0.005	0.009	**0.012**

*p<0.05.

**p<0.01.

***p<0.001.

‡ Plus-minus values are linear regression coefficients and standard errors (i.e., ±SE).

PM_2.5_ – Airborne particulate matter with aerodynamic diameter <2.5 µm.

As shown in the correlation matrix for a 7-day model (**Table 3**), both apparent temperature and air pollution are in a negative correlation with all-season mortality and in a weak to moderate positive correlation between each other (r = 0.42, p<0.001). These analytical results suggest the presence of multicollinearity between the two predictor variables (i.e., apparent temperature vs. air pollution), which provides a plausible explanation for the observed, seemingly paradoxical, negative correlation between air pollution and mortality. When compared to the summer season, air pollution appears to be generally lower in winter (**Figure 3**), coinciding with lower apparent temperatures and higher mortality rates.

**Table 3:** Correlation matrix for all-season mortality, apparent temperature and air pollution (PM_2.5_).

7-Day Model Correlation Matrix	C&R/100K	PM_2.5_ (µg/m^3^)	AT (°C)
**C&R/100K**	**1**		
**PM_2.5_** (µg/m^3^)	−0.11***	**1**	
**AT** (°C)	−0.61***	0.42***	**1**

***p<0.001.

C&R/100K – Mortality from circulatory and respiratory causes per 100K population.

PM_2.5_ – Airborne particulate matter with aerodynamic diameter <2.5 µm.

AT – Apparent Temperature.

Cold season models for apparent temperature below 10°C vs. C&R mortality show increasing prediction reliability with longer time-intervals (r^2^ = 0.24, p<0.001 in a 7-day model (**Table 4**)). On the other hand, warm season models for AT above 15°C indicate that a 3-day model (r^2^ = 0.05, p<0.01) offers the best prediction of heat-related mortality in the studied elderly population (**Table 5**).

**Table 4:** Linear regression estimates of cold season mortality from circulatory and respiratory causes in Metro Vancouver elderly population associated with apparent temperature.

Cold Season (AT<10°C)Linear Regression Coefficient/Variable‡	1-Day Model	2-Day Model	3-Day Model	5-Day Model	7-Day Model
Y-axis Intercept	5.73±0.06***	11.46±0.09***	17.21±0.12***	28.73±0.17***	40.30±0.21***
AT (°C)	−0.08±0.01***	−0.17±0.02***	−0.25±0.02***	−0.43±0.03***	−0.63±0.04***
Observations Count	730	722	721	723	719
Coefficient of Determination (**r^2^**)	0.06	0.11	0.14	0.19	**0.24**

***p<0.001.

‡ Plus-minus values are linear regression coefficients and standard errors (i.e., ±SE).

AT – Apparent Temperature.

**Table 5:** Linear regression estimates of warm season mortality from circulatory and respiratory causes in Metro Vancouver elderly population associated with apparent temperature.

Warm Season (AT>15°C)Linear Regression Coefficient/Variable‡	1-Day Model	2-Day Model	3-Day Model	5-Day Model	7-Day Model
Y-axis Intercept	2.98±0.96**	5.94±1.42***	9.06±1.90***	17.64±2.68***	27.58±3.53***
AT (°C)	0.10±0.05	0.20±0.08*	0.29±0.11**	0.35±0.16*	0.33±0.21
Observations Count	138	135	131	129	123
Coefficient of Determination (**r^2^**)	0.02	0.04	**0.05**	0.04	0.02

*p<0.05.

**p<0.01.

***p<0.001.

‡ Plus-minus values are linear regression coefficients and standard errors (i.e., ±SE).

AT – Apparent Temperature.

## Discussion

### Apparent temperature

**Figure 4:**
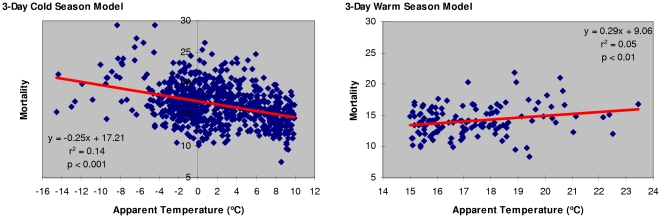
Mortality per 100K elderly population from circulatory and respiratory causes associated with apparent temperature (3-day cold season vs. 3-day warm season model).

**Figure 5:**
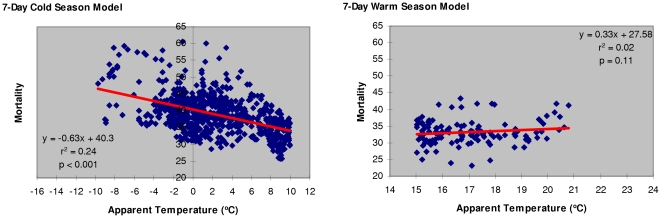
Mortality per 100K elderly population from circulatory and respiratory causes associated with apparent temperature (7-day cold season vs. 7-day warm season model).

Comparative analyses of cold vs. warm season models illustrate the presence of a V-shaped relationship between temperature and mortality (**Figure 4**, **Figure 5**). The analyses reveal that cold winter temperatures are better than warm summer temperatures in predicting elderly mortality, showing an increasing strength for longer time intervals. Approximately 24% of the variation in elderly population mortality from circulatory and respiratory causes in cold season could be accounted for by the variation in a 7-day moving average AT. The results from warm temperature models indicate that a 3-day moving average AT is the best predictor of heat-related mortality, which is in agreement with the published epidemiological evidence [7].

In a changing climate, a 1°C increase of a 3-day moving average AT during summer is associated with 0.29 additional deaths per 100,000 elderly population, which could be translated into ∼27 additional deaths (95%CI: 7.36 to 46.92) during a hypothetical 92-day warm season from June to August in a 300,000 elderly population of Metro Vancouver. Conversely, a cold season 3-day moving average AT decrease of 1°C could lead to 0.25 additional deaths per 100,000 elderly population (i.e., up to 23 additional deaths (95%CI: 19.32 to 27.60) during a hypothetical 92-day cold season). The results suggest that, in the event of a projected global warming [23], an increase in population mortality due to higher temperatures in summer season could be offset by an equivalent reduction in population mortality during warmer than usual conditions in winter season. It should be observed, however, that a 3-day warm season model (r^2^ = 0.05, p<0.01) is less reliable in predicting mortality than 3-, 5-, and 7-day cold season models (r^2^ = 0.14, r^2^ = 0.19, and r^2^ = 0.24, p<0.001, respectively).

### Air pollution

A 7-day model multiple correlation analysis for AT and PM_2.5_ vs. all-season C&R mortality shows an adjusted multiple coefficient of determination R^2^ = 0.39 (p<0.001), with univariate contributions of approximately 37% from AT (p<0.001) and only 1% from PM_2.5_ (p<0.001). Hence, rather than air pollution, elderly mortality is more likely to be associated with the variation in apparent temperature.

Metro Vancouver has been generally considered as a city with low levels of air pollution when compared to other metropolitan areas of North America [24], [25]. On the basis of data presented in this paper, the mean daily PM_2.5_ concentration in Metro Vancouver for the period from 2004 to 2006 was approximately 5.27 µg/m^3^, (min. = 0.61 µg/m^3^; max. = 31.02 µg/m^3^). Although high concentrations of fine airborne particulate matter, typically approaching or exceeding 50 µg/m^3^ for PM_2.5_ or 100 µg/m^3^ for PM_10_ in heavily polluted and industrial cities, could be expected to show an association with adverse health effects and increased mortality [11], [12], [26], there is no universal agreement among scientists that low-level air pollution at concentrations close to the current Canadian or US Ambient Air Quality guidelines for PM_10_ and PM_2.5_ affect significantly human health and cause increased mortality [27], [28].

It has been proposed that there is no threshold concentration for particulate matter pollution, below which no increased incidence of mortality could be observed for cardiovascular and respiratory causes [14], [29], [30]. However, as suggested by Vedal et al. (2003), the findings that health and mortality effects are observed even at low levels of fine particulate matter air pollution “*may support the notion that no threshold pollutant concentrations are present, but they also raise concern that these effects may not be effects of the measured pollutants themselves, but rather of some other factor(s) present in the air pollution-meteorology mix*” (emphasis added) [25]. Compared to low-level air pollution, the seasonal variation in meteorological conditions and influenza outbreaks are expected to have overwhelmingly stronger effects on mortality and population health. This may render our ability to measure PM_10_- or PM_2.5_-attributable health impacts increasingly more difficult and less reliable in metropolitan areas with low levels of air pollution, such as Metro Vancouver.

### Confounding factors and uncertainties

There is a seasonal variation and a strong correlation between air temperature and insolation [31], [32]. The reduced insolation and erythemal UV-B irradiation in winter periods is associated with lower generation of vitamin D in the exposed skin and higher probability for developing vitamin D insufficiency/deficiency [33], [34]. As vitamin D is a recognized modulator of the immune response [35], insufficiency/deficiency in this important micronutrient may lead to a reduced capacity of the immune system to respond to infections or acute/chronic inflammatory conditions. Vitamin D deficiency has been associated with the adverse effects on respiratory health [36], vascular inflammation, and cardiovascular disease [37]. Hence, incremental contribution of vitamin D deficiency to the observed temperature-associated mortality rates during cold season cannot be ruled out on the basis of the results presented in this paper.

Possible effects of socio-economic status, body mass index, smoking status, gender differences, demographic structure, ethnic origin, air pollution “hot spots” [38] and variability in micro-climatic conditions in the studied geographical area are not included or controlled for in the analyses. Further research and a more comprehensive study is needed to account for the effects from these and other potential confounding factors, and to refine the observed associations between the studied variables.

### Conclusions

Apparent temperature is associated with mortality from circulatory and respiratory causes, while air pollution does not appear to be a reliable predictor of elderly population mortality on the regional level in Metro Vancouver. All-season and cold temperature models show increasing prediction reliability with longer time-intervals, and a 3-day model offers the best prediction of heat-related mortality. The results presented in this paper suggest that, in a changing climate, one should take into consideration potential health impacts from the projected high- as well as low-temperature extremes, keeping in mind that temperature-attributable population mortality rates peak in cold season.
